# Reconfigurable Amphiphilic DNA Nanotweezer for Targeted Delivery of Therapeutic Oligonucleotides

**DOI:** 10.1021/acscentsci.4c01152

**Published:** 2024-12-05

**Authors:** Shuxuan Shao, Wei Du, Shuang Liu, Canqiong Hu, Cao Zhang, Lexun Li, Fan Yang, Qiaoling Liu, Weihong Tan

**Affiliations:** † Molecular Science and Biomedicine Laboratory (MBL), State Key Laboratory of Chemo/Biosensing and Chemometrics, FuRong Laboratory, College of Biology, Hunan University, Changsha, Hunan 410082, China; ‡ Department of Pathology, Changde Hospital, Xiangya School of Medicine, Central South University (The First People’s Hospital of Changde City), Changde, Hunan 415000, China; § The Cancer Hospital of the University of Chinese Academy of Sciences (Zhejiang Cancer Hospital), Institute of Basic Medicine and Cancer (IBMC), Chinese Academy of Sciences, Hangzhou, Zhejiang 310022, China; ∥ School of Materials Science and Engineering, Institute of Molecular Medicine (IMM), Renji Hospital, Shanghai Jiao Tong University School of Medicine, Shanghai Jiao Tong University, Shanghai 200240, China

## Abstract

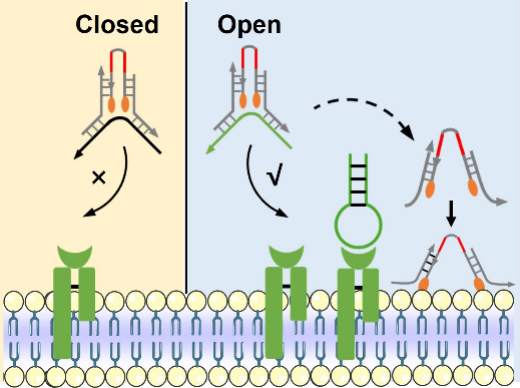

Amphiphilic lipid oligonucleotide conjugates are powerful molecular-engineering materials that have been used for delivery of therapeutic oligonucleotides. However, conventional lipid oligonucleotide conjugates suffer from poor selectivity to target cells due to the nonspecific interaction between lipid tails and cell membranes. Herein, a reconfigurable DNA nanotweezer consisting of a c-Met aptamer and bischolesterol-modified antisense oligonucleotide was designed for c-Met-targeted delivery of therapeutic antisense oligonucleotides. The c-Met aptamer is used to keep the DNA nanotweezer in a “closed” state, which enables the hydrophobic interaction within bischolesterol moieties. As a result, the amphiphilic DNA nanotweezer shows only a weak interaction with the cell membrane. Upon the release of the c-Met aptamer, the DNA nanotweezer converts to an “open” state, which facilitates the insertion of a cholesterol moiety into the cell membrane. Thus, the reconfigurable DNA nanotweezer enables the selective membrane anchoring of the DNA nanotweezer in cancerous cells that highly expressed c-Met protein. Moreover, this amphiphilic DNA nanotweezer shows enhanced accumulation at the tumor site and the inhibition of tumor growth. Taking advantage of the stimuli-responsive membrane anchoring capability, this reconfigurable DNA nanotweezer could be further explored as a smart multifunctional platform for cancer therapy.

## Abstract 2

c-Met protein-induced conformational transition of the amphiphilic DNA nanotweezer from “closed” to “open” state enables the tunable membrane anchoring capability for enhanced targeted delivery.

## Introduction

Lipid oligonucleotide conjugates are promising therapeutic agents in biomedical applications due to their excellent properties, including strong cellular permeability,^1^ long circulation time,^2,3^ and great physiological stability.^4,5^ Until now, a multitude of lipid oligonucleotide conjugates have been tested in clinical trials for the treatment of cancer, skin disease, and other diseases.^6^ For instance, ARC-520-HBV, a lipid oligonucleotide composed of a pair of cholesterol-conjugated siRNAs has been used in the treatment for hepatitis B virus (HBV) in phase II.^7^ RXI-109, a cholesterol-conjugated siRNA with positive results in phase II clinical trials for reducing scar formation.^8^ Likewise, GRN163L is a palmitic acid conjugate antisense oligonucleotide that has demonstrated an inhibitory activity against multiple myeloma in phase I clinical trials.^9^ These encouraging clinical results verified the effectiveness of the lipid oligonucleotide conjugates in disease treatment.

Despite these advances, the therapeutic lipid oligonucleotide conjugates suffer from poor selectivity to the target cell.^10,11^ Since the mammalian cell membranes share similar phospholipid bilayer structure, the nonselective hydrophobic interaction between the lipid tail and the cell membrane occurs during the delivery of therapeutic lipid oligonucleotide conjugates.^11,12^ To address this issue, researchers designed novel lipid oligonucleotide conjugates by chemically modifying the lipid moiety to improve their selectivity to target cells. For example, Jin et al. synthesized a phosphorylated lipid-conjugated oligonucleotide (DNA-lipid-P) for alkaline phosphatase (ALP)-dependent cell targeting. In the presence of ALP, DNA-lipid-P converted to DNA-lipid with a greater hydrophobicity by enzyme-catalyzed dephosphorylation. Thus, the *in situ* conversion of DNA-lipid-P on the membrane surface facilitated the selective binding of DNA-lipid to cells with high levels of alkaline phosphatase.^11^ However, the chemical modification of lipid moiety constantly needs multistep chemical reactions with complex procedures and poor overall yields, which limits their application.^13,14^ Alternatively, lipid-based DNA micelles formed by hydrophobic self-assembly from lipid-conjugated DNA are used to avoid the nonspecific insertion of lipid moiety into the cell membrane.^15^ To improve the targeting capability of lipid-DNA micelles, recognition molecules (e.g., aptamer, folate) have been introduced into the micelles.^16,17^ However, these DNA micelles could lose their functionality due to their structural instability in complex physiological environments, which hinders their application *in vivo*.^18^ Therefore, the development of a facile strategy to improve the targeting ability of lipid oligonucleotide conjugates is still in great demand.

DNA nanostructures modified with multiple cholesterol anchors can have strong tendencies to aggregate by way of hydrophobic interactions, which reduces membrane anchoring efficiency.^19,20^ Thus, tuning the hydrophobic interactions in proximal cholesterol molecules could modulate the accessibility of cholesterol anchors to the cell membrane, which in turn, controls the targeting capability of lipid oligonucleotide conjugates.^21^ As a switchable DNA nanodevice with elegant simplicity, a DNA nanotweezer has the structural flexibility required for tuning the interactions of cholesterols attached to the ends of the tweezers.^22,23^ A DNA nanotweezer can change its conformation from “closed” to “open” by a toe-hold-mediated DNA strand displacement reaction, which causes the variation of cholesterols’ spacing and achieves the modulation of their hydrophobic interactions.^24,25^

**Figure 1:**
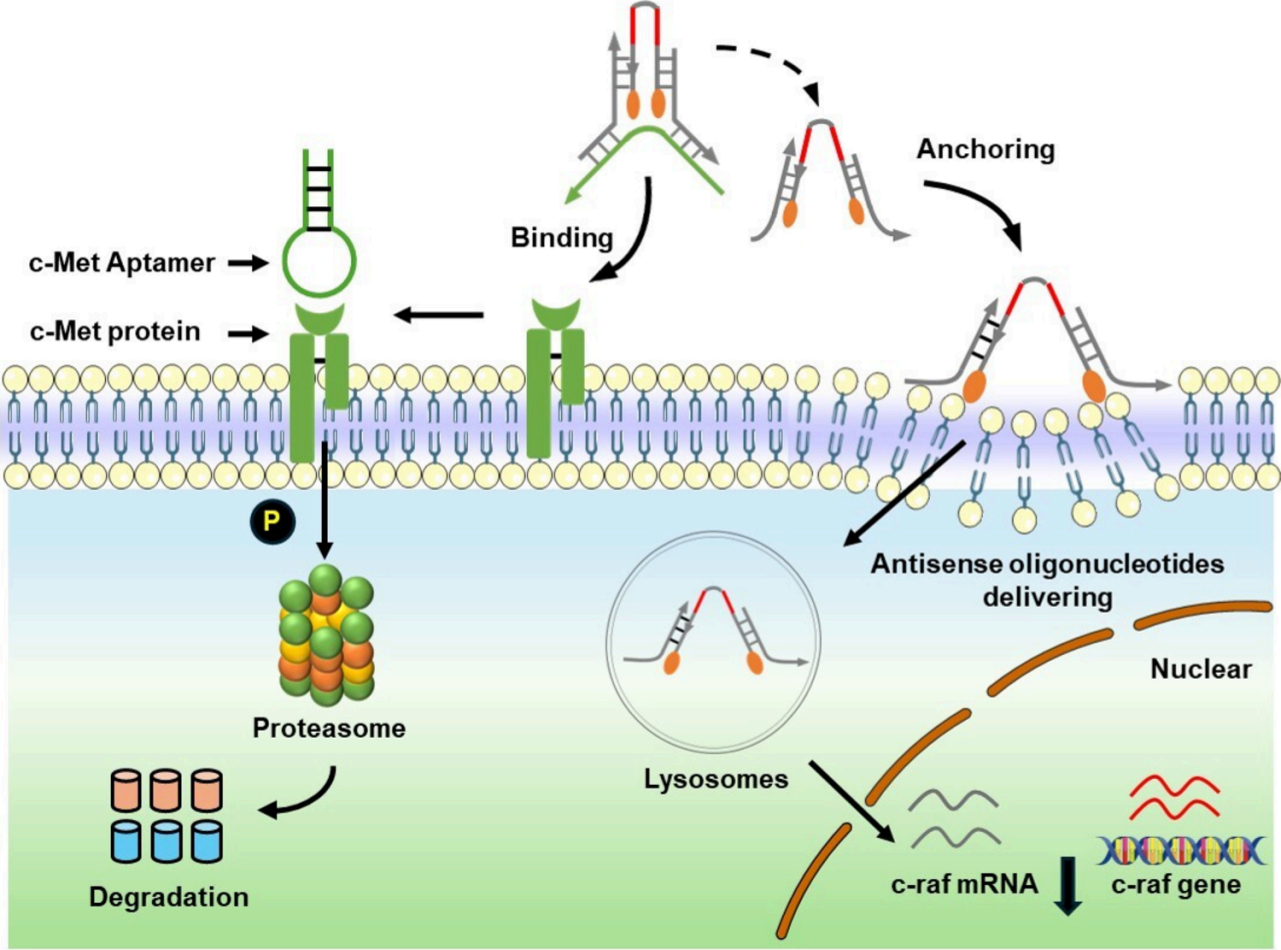
Schematic illustration of a reconfigurable amphiphilic DNA nanotweezer for targeted delivery of antisense oligonucleotides.

In this work, we designed a reconfigurable amphiphilic DNA nanotweezer composed of a c-Met aptamer and bischolesterol-modified antisense oligonucleotide (ASO) (Figure 1). The c-Met aptamer was introduced as the molecular recognition unit to dynamically regulate the conformation change of the amphiphilic DNA nanotweezer. In the absence of c-Met protein, the DNA nanotweezers kept “closed” state and showed only weak interaction with the cell membrane due to the bischolesterol-endowed intramolecular hydrophobic interaction. Conversely, the DNA nanotweezer was converted to “open” state and could be easily anchored to the cell membranes with high c-Met expression. As a result of such conversion, the ASO could be effectively transported into target cells and effectively inhibit tumor cell growth and migration. Our work provides a potential avenue for developing lipid oligonucleotide conjugates with excellent targeting ability, which sheds light on broadening their biomedical applications in cancer therapy.

## Results and Discussion

### Construction and Characterization of Amphiphilic DNA Nanotweezer with Tunable Membrane Anchoring Capability

**Figure 2:**
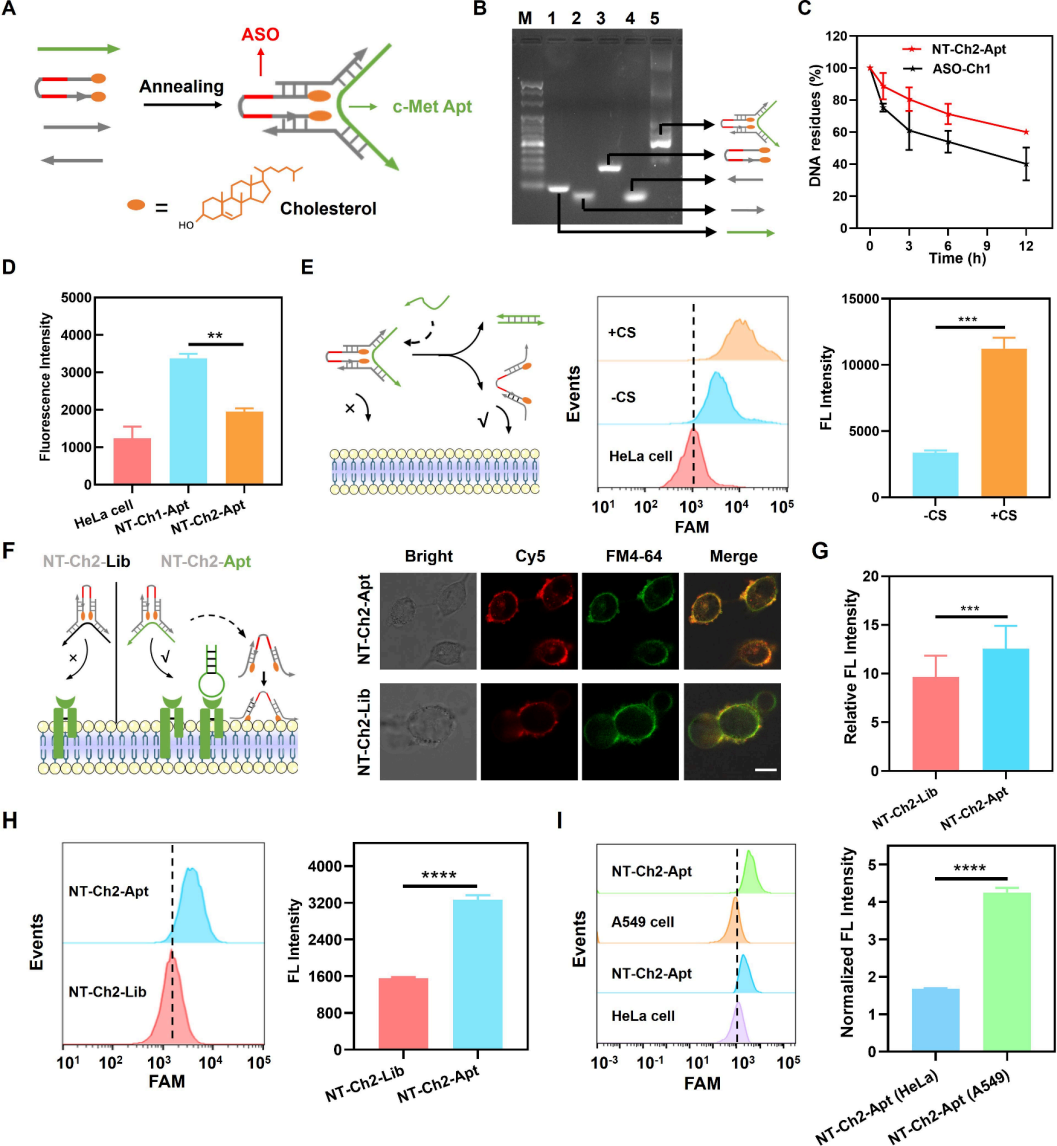
Construction and characterization of an amphiphilic DNA nanotweezer. (A) Schematic diagram of a bischolesterol-modified DNA nanotweezer (NT-Ch2-Apt). (B) 3% agarose gel electrophoresis assay of NT-Ch2-Apt. (C) The degradation analysis of NT-Ch2-Apt and ASO-Ch1 in RPMI1640 medium supplemented with 10% FBS. (D) Flow cytometry analysis of HeLa cells incubated with NT-Ch1-Apt and NT-Ch2-Apt for 0.5 h separately. (E) Flow cytometry analysis of HeLa cells incubated with NT-Ch2-Apt for 0.5 h in the presence of complementary strand (+CS) or the absence of complementary strand (−CS). (F) Confocal images of A549 cells incubated with Cy5-labeled NT-Ch2-Lib and Cy5-labeled NT-Ch2-Apt separately. Cell membrane was stained by FM4-64 dye. Scale bar, 10 μm. (G) Quantification of the relative fluorescence intensity of Cy5 channel in different treatment groups in (F). Results are presented as means ± standard deviation (SD) (*n* = 20). (H) Flow cytometry analysis of A549 cells incubated with NT-Ch2-Lib and NT-Ch2-Apt for 0.5 h separately. (I) Flow cytometry analysis of HeLa cells and A549 cells incubated with NT-Ch2-Apt for 0.5 h separately. Normalized fluorescence intensity was shown on the right. Results are presented as means ± standard deviation (SD) (*n* = 2 for C–E; *n* = 3 for H–I). **P ≤ 0.01, ***P ≤ 0.001, and ****P ≤ 0.0001 by two-tailed Student’s *t* test.

As illustrated in Figure 2A, the amphiphilic DNA nanotweezer consists of a c-Met aptamer, a bischolesterol-conjugated ASO (c-raf-1), and two arm strands. The two arm strands of the tweezer are held together by the c-Met aptamer, and the bicholesterol is in close contact due to the proximity of the cholesterol moieties attached at the end of ASO. We named this amphiphilic DNA nanotweezer NT-Ch2-Apt. Similarly, an amphiphilic DNA nanotweezer with monocholesterol-conjugated ASO was named NT-Ch1-Apt. The agarose gel electrophoresis analysis confirms the self-assembly of amphiphilic DNA nanotweezer as designed (Figures 2B and S1). The NT-Ch2-Apt exhibits good stability as compared with monocholesterol-conjugated ASO (ASO-Ch1) in the culture medium supplemented with 10% FBS for 12 h (Figures 2C and S2). To confirm the conformation change of NT-Ch2-Apt,^24^ we labeled the 5′ -end and 3′ -end of the ASO strand with the fluorophore FAM and the quencher BHQ1, separately. DNA nanotweezers showed weak fluorescence intensity in “closed” state due to the proximity of fluorophore FAM and quencher BHQ1. Upon the addition of a complementary strand of c-Met aptamer, the toe-hold mediated strand displacement of c-Met aptamer leads to the fluorescence intensity recovery of FAM dye (Figures S3 and S4), indicating the conformation change of DNA nanotweezer from “closed” to “open”. As a control, the addition of the noncomplementary strand of c-Met aptamer displayed only low fluorescence signals (Figures S5 and S6). Moreover, the gel electrophoresis analysis verified the rapid switch of NT-Apt from “closed” to “open” state upon the addition of a complementary strand of c-Met aptamer (Figure S7). These results together confirmed the tunable conformation of the designed DNA nanotweezer.

To assess the membrane anchoring capability of the amphiphilic DNA nanotweezer, nontarget HeLa cells were incubated with the amphiphilic DNA nanotweezer and analyzed by flow cytometry. As shown in Figure 2D, there was a significant difference in fluorescence intensity of HeLa cells treated with NT-Ch1-Apt and NT-Ch2-Apt respectively, indicating the lower membrane accessibility of NT-Ch2-Apt than that of NT-Ch1-Apt. We further tested the tunable membrane anchoring capability of NT-Ch2-Apt by the strand displacement reaction. As shown in Figure 2E, in the presence of the complementary strand of c-Met aptamer, the toe-hold mediated strand displacement reaction leads to the “open” state of NT-Ch2-Apt, and an obvious fluorescence signal could be detected from HeLa cells. These results suggested that the membrane anchoring of NT-Ch2-Apt can be effectively modulated by releasing the c-Met aptamer from the amphiphilic DNA nanotweezer.

To investigate whether the binding of the c-Met aptamer with c-Met protein could facilitate the target protein-triggered membrane anchoring of the DNA nanotweezer, A549 lung cancer cells with highly expressed c-Met protein were used. As shown in Figure 2F, 2G, and 2H, the specific binding of c-Met aptamer to c-Met protein leads to the anchoring of NT-Ch2-Apt on the membrane surface of A549 cells. To further verify the c-Met aptamer-mediated specific response of A549 cells to NT-Ch2-Apt, c-Met siRNA-treated A549 cells were used. As expected, the fluorescence intensity in c-Met knockdown A549 cells incubated with NT-Ch2-Apt was similar to that of intact A549 cells incubated with arbitrary library guided-NT-Ch2 (NT-Ch2-Lib) (Figure S8). Besides, HeLa cells with a low c-Met protein on the membrane surface were used as the control (Figure S9). As shown in Figure 2I, the normalized fluorescence intensity of A549 cells incubated with FAM-labeled NT-Ch2-Apt is 2.5-fold higher than that of HeLa cells. Meanwhile, nontarget HeLa cells showed low fluorescence intensity and without a significant difference upon the treatment with either NT-Ch2-Apt or NT-Ch2-Lib (Figure S10). Taken together, the reconfigurable amphiphilic DNA nanotweezer showed good performance on selective membrane anchoring, which facilitates the targeted delivery of ASO.

### Amphiphilic DNA Nanotweezer Could Efficiently Inhibit Cell Proliferation and Migration

**Figure 3:**
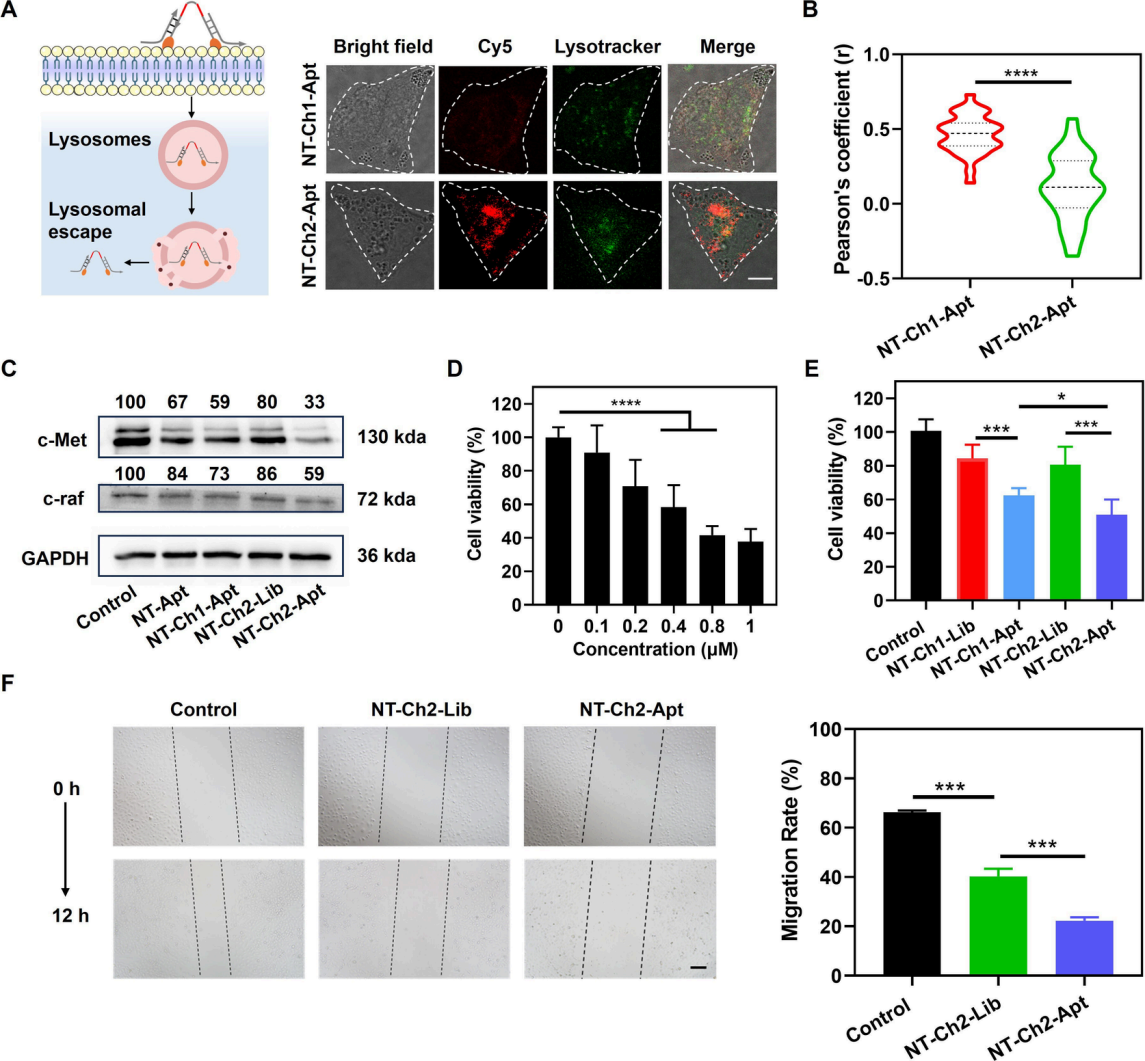
Cellular internalization of NT-Ch2-Apt and down-regulation of expression of protein. (A) Confocal images of A549 cells incubated with NT-Ch1-Apt and NT-Ch2-Apt separately. The colocalization of Cy5-labeled nanotweezer and LysoTracker Green was shown. Scale bar, 10 μm. (B) The Pearson’s correlation coefficients of Cy5-labeled DNA nanotweezer versus LysoTracker Green-labeled lysosomes. (C) Western blot analysis of c-Met and c-raf protein in A549 cells treated with NT-Apt, NT-Ch1-Apt, NT-Ch2-Lib, and NT-Ch2-Apt separately for 24 h. Numbers in (C) were the relative values determined by the gray value of the target protein band/gray value of GAPDH. (D) Cell viability assay of A549 cells incubated with NT-Ch2-Apt at different concentrations (0–1 μM). (E) Cell viability of A549 cells incubated with different DNA nanotweezers (NT-Ch1-Lib, NT-Ch1-Apt, NT-Ch2-Lib, and NT-Ch2-Apt) separately for 48 h. (F) *In vitro* cell scratch healing assay of A549 cells incubated with NT-Ch2-lib and NT-Ch2-Apt separately for 12 h. Scale bar, 50 μm. *P ≤ 0.05, **P ≤ 0.01, ***P ≤ 0.001, and ****P ≤ 0.0001 by two-tailed Student’s *t* test.

Lipid oligonucleotide conjugates anchored on the membrane surface could be entered into cells by endocytosis.^26^ The time-dependent enhancement of the fluorescence intensity in A549 cells indicated the cellular uptake of NT-Ch2-Apt (Figure S11). NT-Ch2-Apt showed more than 3-fold higher cellular fluorescence intensity than that of NT-Ch1-Apt. Once entered the cell, NT-Ch2-Apt showed apparent endosomal escape (Figures 3A and S12), as the Pearson’s correlation coefficient of lysotracker and Cy5-labeled NT-Ch2-Apt is significantly lower than that of lysotracker and Cy5-labeled NT-Ch1-Apt (Figure 3B). It has been reported that the lipid moiety could affect endosomal membrane stability through enhanced interaction with endosomal membranes, resulting in the efficient escape of lipid oligonucleotide conjugates from endosomes.^27^ NT-Ch2-Apt may affect endosomal membrane stability by enhancing the interaction with endosomal membrane, thereby leading to efficient lysosomal escape.

We then evaluated the therapeutic effect of NT-Ch2-Apt in the A549 cells. As we know, c-Met aptamer could induce the ubiquitination and degradation of c-Met protein via molecular recognition-triggered,^28,29^ while c-raf-1 ASO could down-regulate the expression of c-raf-1.^30^ Thus, the degradation of c-Met protein and downregulation of c-raf-1 would synergistically inhibit the downstream of Ras-Raf-MAPK signaling pathways and suppress the invasion and migration of cancer cells.^31^ As expected, for the A549 cells treated with NT-Ch2-Apt, the Western blot analysis demonstrated the reduction of approximately 67% and 41% of c-Met protein and c-raf protein respectively (Figures 3C and S13). Moreover, NT-ASO-Apt, NT-ASO-Lib (DNA nanotweezers with nontarget library sequences), NT-SC-Apt (DNA nanotweezers with scrambled ASO sequences), and NT-SC-Lib (DNA nanotweezer with scrambled ASO sequence and nontargeted library sequence) were used to verify the synergistic inhibition of the downstream of Ras-Raf- MAPK signaling pathways by DNA nanotweezer composed of c-raf ASO and c-Met aptamers. As shown in Figure S14, A549 cells treated with NT-ASO-Apt showed the strongest down-regulation effect on c-Met and c-Raf compared with other treatment groups. The downregulation of c-raf-1 protein and the degradation of c-Met protein lead to decreased cell viability and migration. As shown in Figure 3D, the cell viability analysis by the Cell Counting Kit-8 (CCK-8) displayed a dose-dependent killing effect of NT-Ch2-Apt. Compared with other amphiphilic DNA nanostructures, NT-Ch2-Apt showed a significant difference in cell viability inhibition on A549 cells (Figures 3E and S15). Additionally, NT-Ch2-Apt showed negligible cytotoxicity to nontarget HeLa cells at concentrations ranging from 100 to 800 nM (Figure S16). In a scratch-healing experiment, the scratch gap in the NT-Ch2-Apt group was much larger than that in the NT-Ch2-Lib group (Figure 3F). The healing rate of the NT-Ch2-Apt group was 22.3%, much lower than that of the PBS group (66.3%) and NT-Ch2-Lib group (40.3%). Collectively, the results demonstrated that NT-Ch2-Apt could efficiently inhibit the proliferation and migration of A549 cells by the targeted delivery of therapeutic ASO.

### *In Vivo* Biodistribution of NT-Ch2-Apt and the Inhibitory Effect on Tumor Growth

**Figure 4:**
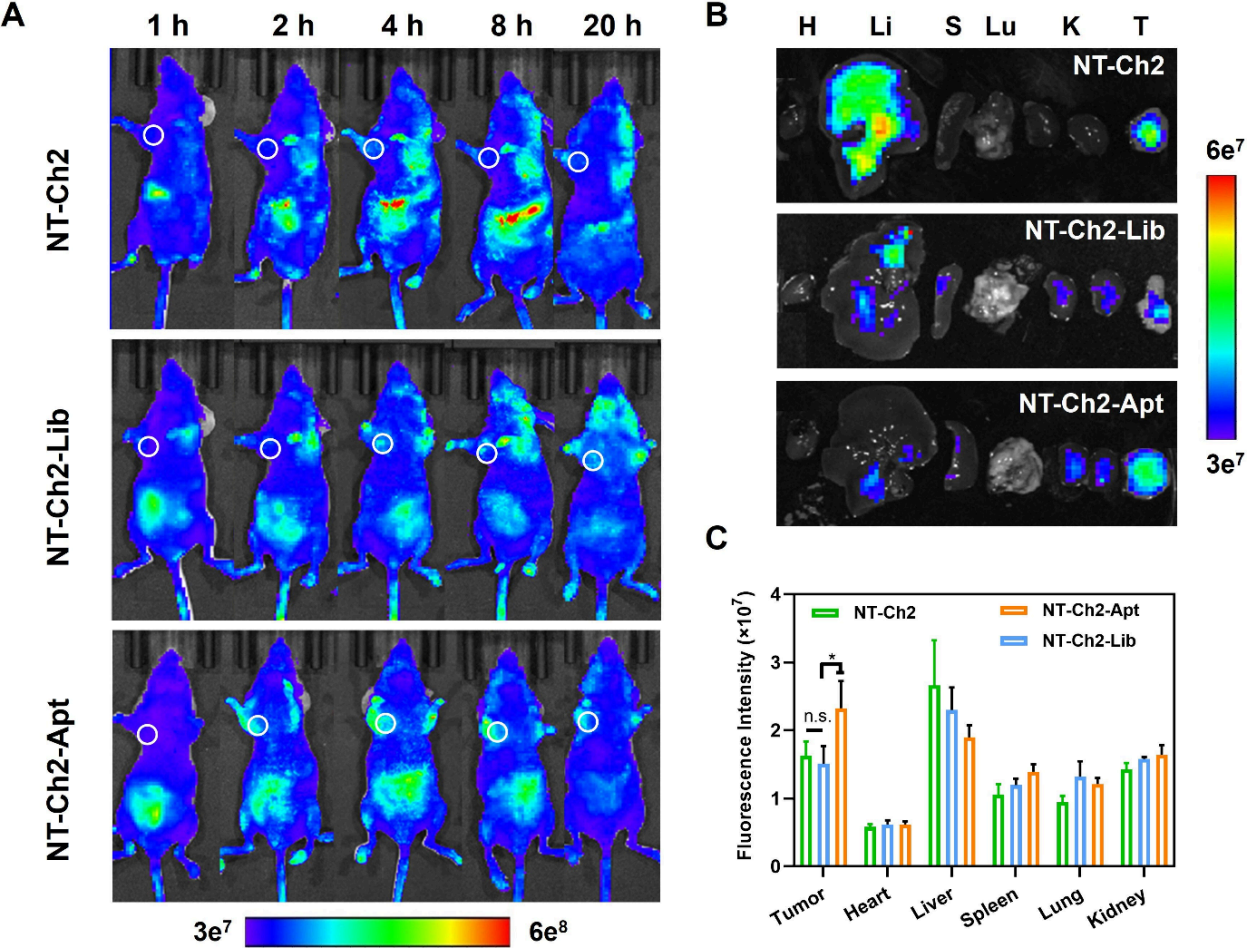
*In vivo* biodistribution of amphiphilic DNA nanotweezer. (A) The fluorescence imaging of A549 tumor-bearing mice at different time intervals after intravenous administration of Cy5-labeled NT-Ch2, Cy5-labeled NT-Ch2-Lib, and Cy5-labeled NT-Ch2-Apt separately. (B) The fluorescence images of *ex vivo* organs after 20 h postinjection (H: heart; Li: liver; S: spleen; Lu: lung; K: kidney) and tumor (T). (C) The quantitative analysis of the averaged fluorescence intensities of the dissected organs and tumors. All statistical data are presented as the mean value ± S.D., *n* = 3. *P ≤ 0.05 by two-tailed Student’s *t* test.

The *in vitro* targeting ability and inhibitory effect of NT-Ch2-Apt on tumor cells prompted us to further explore its performance *in vivo*. We first investigated the biodistribution of NT-Ch2-Apt in A549 tumor-bearing nude mice by fluorescence imaging. As shown in Figure 4A, NT-Ch2-Apt predominantly accumulated in the tumor site after tail vein injection. In contrast, a weak fluorescence signal was observed in tumor sites for nontargeted NT-Ch2 and NT-Ch2-Lib. The *ex vivo* fluorescence imaging of the major organs and tumor tissues demonstrated that NT-Ch2-Apt has the highest fluorescence signal in anatomic tumor (Figure 4B). Quantitative fluorescence analysis also revealed that the accumulation of NT-Ch2-Apt in tumor sites was significantly higher than that in other groups (Figure 4C), which corroborated the targeting capability of NT-Ch2-Apt in A549 nude mice.

**Figure 5:**
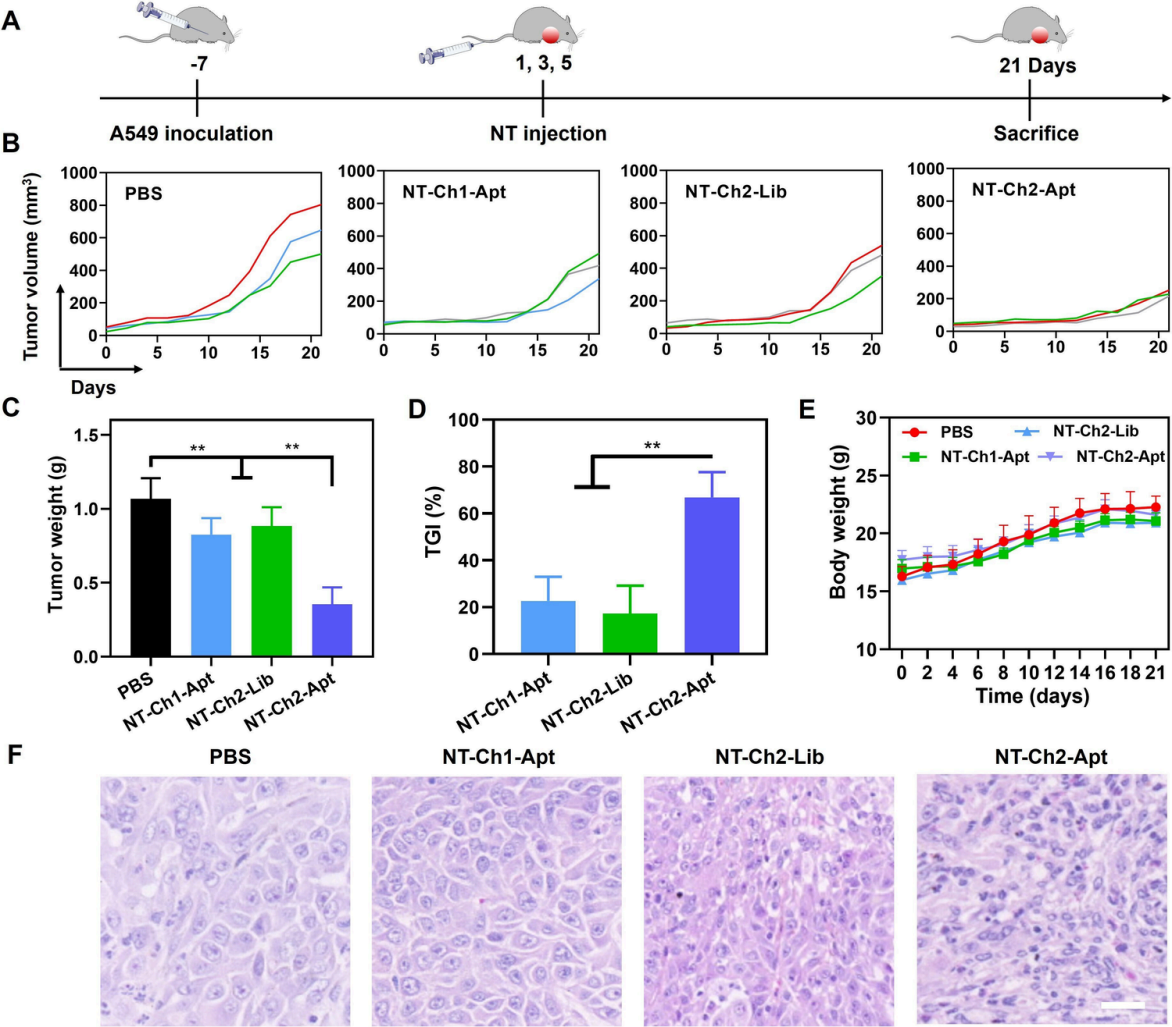
*In vivo* tumor growth inhibition by amphiphilic DNA nanotweezer. (A) Schematic diagram of the treatment procedures for the A549 tumor-bearing mice. (B) Tumor growth profiles of A549 tumor-bearing mice after the treatment with PBS, NT-Ch1-Apt, NT-Ch2-Lib, and NT-Ch2-Apt separately. (C) Tumor weight evaluation after the treatment with different groups. (D) Tumor growth inhibition (TGI) ratio of A549 tumor-bearing mice after the indicated treatment. (E) Body weight of A549 tumor-bearing mice with indicated treatment during 21 days. (F) Representative H&E staining images of the tumor tissues after the indicated treatment. Scale bar, 50 μm. All statistical data are presented as the mean value ± S.D., *n* = 3. **P ≤ 0.01 by two-tailed Student’s *t* test.

To investigate the therapeutic potential of NT-Ch2-Apt, A549 tumor-bearing nude mice were treated with different amphiphilic DNA nanotweezers separately, and the tumor volume and body weight were recorded every 2 days until the end of treatment on day 21 (Figure 5A). As shown in Figure 5B, the tumors grew substantially slowly in mice treated with NT-Ch2-Apt. In contrast, mice treated with NT-Ch1-Apt or NT-Ch2-Lib formed fast-growing tumors. The NT-Ch2-Apt group exhibited an obvious antitumor effect with an approximate 60% reduction in tumor weight, while the NT-Ch1-Apt and NT-Ch2-Lib groups showed a reduction in tumor weight of approximately 35% and 30%, respectively (Figures 5C, S17). The tumor growth inhibition (TGI) ratio also confirmed the excellent therapeutic effect of NT-Ch2-Apt in inhibiting tumor growth (Figure 5D). The treatment of NT-Ch2-Apt did not cause additional weight loss compared to PBS treatment in A549 tumor-bearing nude mice (Figure 5E). Moreover, the H&E image analysis revealed normal histological architecture in major organs but a high level of apoptosis in tumor tissue, further confirming the antitumor efficacy of NT-Ch2-Apt *in vivo* (Figures 5F and S18). These results clearly showed that the NT-Ch2-Apt can inhibit tumor progression effectively through the targeted delivery of therapeutic ASO.

## Conclusions

In summary, we constructed a reconfigurable amphiphic DNA nanotweezer for targeted delivery of antisense oligonucleotide. This structurally well-defined delivery system with tunable conformation facilitates selective anchoring of the DNA nanotweezer on the target cell membrane. The NT-Ch2-Apt enables efficient cellular uptake and subsequent endosomal escape, which leads to the synergistic down-regulation of the expression of c-raf-1 protein and the degradation of c-Met protein, as well as the inhibition of cell proliferation and mitigation. Moreover, the accumulation of NT-Ch2-Apt in tumor sites promoted its antitumor effects *in vivo*. Overall, our study represents a promising approach to improving the targeting ability of lipid oligonucleotide conjugates and highlights the potential of NT-Ch2-Apt as an excellent platform for the delivery of therapeutic oligonucleotides.

## Supporting Information Available

Experimental section; oligonucleotide sequence tables (Table S1); and supporting figures (Figures S1–S18) (PDF)
