# Supplementary material for: Reconfigurable Amphiphilic DNA Nanotweezer for Targeted Delivery of Therapeutic Oligonucleotides
Source: ACS Cent Sci. 2024 Dec 5;10(12):2338–45. doi: 10.1021/acscentsci.4c01152 (PMC11672532; doi:10.1021/acscentsci.4c01152)
Supplement: Supplementary file 1 — oc4c01152_si_001.pdf [file oc4c01152_si_001.pdf]

Supporting Information

Reconfigurable amphiphilic DNA nanotweezer for targeted delivery of therapeutic oligonucleotides

Shuxuan Shao [a], *Wei Du* [b], *Shuang Liu* [a], *Canqiong Hu* [a], *Cao Zhang* [a], *Lexun Li* [a], *Fan Yang* [a], *Qiaoling Liu* [a*], *Weihong Tan* [a, c, d]

[a] Molecular Science and Biomedicine Laboratory (MBL), State Key Laboratory of Chemo/Biosensing and Chemometrics, FuRong Laboratory, College of Biology, Hunan University, Changsha, Hunan 410082, China.

[b] Department of Pathology, Changde Hospital, Xiangya School of Medicine, Central South University (The First People's Hospital of Changde City), Changde, Hunan, 415000, China.

[c] The Cancer Hospital of the University of Chinese Academy of Sciences (Zhejiang Cancer Hospital), Institute of Basic Medicine and Cancer (IBMC), Chinese Academy of Sciences, Hangzhou, Zhejiang 310022, China.

[d] School of Materials Science and Engineering, Institute of Molecular Medicine (IMM), Renji Hospital, Shanghai Jiao Tong University School of Medicine, Shanghai Jiao Tong University, Shanghai 200240, China.

Contents

1. Reagents and materials 3
2. Experimental section 3
Cell lines and cell culture 3
Preparation of amphiphilic DNA nanotweezer..... 4
Stability analysis of NT-Ch2-Apt and ASO-Ch1..... 4
Gel electrophoresis..... 4
Fluorescence measurements 5
Flow cytometry assay..... 5
Confocal microscope imaging 6
Cell viability assay 7
c-Met siRNA knockdown experiment 7
Western blot analysis..... 8
Cell migration assay..... 9
In vivo tumor imaging.....9
In vivo antitumor activity assay.....10
HE staining analysis of tissue sections.....10
Statistical analysis 11
Safety 11
3. Supplementary Tables..... 12
4. Supplementary Figures..... 13

1. Reagents and Materials

All DNA oligonucleotides were synthesized by Sangon Biotech Co., Ltd. (Shanghai, China) with standard desalting, purified with high-performance liquid chromatography, and used without further purification. c-Met siRNA was purchased from Beijing Tsingke Biotech Co., Ltd. Lipofectamine 3000 was purchased from Invitrogen, USA. Cell Counting Kit-8 (CCK-8) was purchased from Dojindo Molecular Technologies, Inc., (Kumamoto, Japan). 6 × DNA loading buffer and DNA marker were purchased from TaKaRa Biotechnology Co., Ltd. (Dalian, China). 5 × Native Gel sample loading buffer was purchased from Biosharp Biotechnology Co., Ltd. (Hefei, China). LysoTracker Green DND-26 was purchased from Thermo Fisher Scientific, USA. The anti-rabbit GAPDH antibody and anti-rabbit c-Met antibody were purchased from Cell Signaling Technology, USA. Anti-rabbit c-raf antibody was purchased from Bethyl Laboratories Inc, USA. The HRP-conjugated secondary antibody was purchased from Millipore, USA. The Hematoxylin-Eosin Staining Kit was purchased from Solarbio Science & Technology Co., Ltd. (Beijing, China).

2. Experimental section

Cell lines and cell culture

A549 cells (a human lung cancer cell line) were grown in RPMI-1640 medium (Gibco, USA) supplemented with 10 % fetal bovine serum (Gibco, USA). HeLa cells (a human cervical carcinoma cell line) were grown in DMEM medium (Gibco, USA)

supplemented with 10 % fetal bovine serum (Gibco) in a cell culture incubator at 37 °C in a humidified 5 % CO_2 atmosphere.

Preparation of amphiphilic DNA nanotweezer

DNA strands were dissolved in ultrapure water and quantitated by NanoDrop 2000 UV-Vis Spectrophotometer (Thermo Scientific Co., Ltd.). The DNA solutions were diluted to the concentration of 100 μM in 1 × TAE buffer (40 mM Tris, 1 mM EDTA, pH=8). To prepare amphiphilic DNA nanotweezer, well-mixed solutions of DNA arm strand 1 and ASO-Ch2 were heated to 95 °C and kept for 10 min and then cooled to 4 °C in 30 s by TGreat Gradient Thermal Cycler (TIANGEN Biotech Co., Ltd.). Then, the product was reacted with annealed DNA arm strand 2 in a water bath at 37 °C for 1 h, and the obtained DNA triple complex was further reacted with annealed c-Met aptamer in a water bath at 37 °C for 1 h. The sample was placed at 4 °C for use.

Stability analysis of NT-Ch2-Apt and ASO-Ch1

As-prepared NT-Ch2-Apt and ASO-Ch1 were incubated with RPMI-1640 complete medium supplemented with 10 % FBS separately at 37 °C for 0 h, 1 h, 3 h, 6 h and 12 h separately. The DNA residual was detected by 3 % agarose gel electrophoresis and further quantified using the gray value of each polymeric by the Image J analysis.

Gel electrophoresis

3 % agarose gel, stained with ethidium bromide (EB), was used for agarose gel electrophoresis. 10 μL DNA sample and 2 μL 6 × loading buffer were mixed and used

as sample solution. Electrophoresis was performed in fresh 1 × Tris-borate-EDTA buffer (1 × TBE, 90 mM Tris, 90 mM boric acid, and 10 mM EDTA, pH 8.0) at 90 V for 15 min, 110 V for 90 min in an ice-water bath. Tanon multi 5200 (Tanon, China) was used to image and analyze all agarose gels. 20-bp DNA ladder was used. For the characterization of strand displacement reaction of NT-Apt in solution, an equal amount of complementary strand and NT-Apt was incubated in 1 × TAE buffer (40 mM Tris, 1 mM EDTA, pH=8) solution at 37 °C for 15 min, 30 min, 45 min, and 60 min separately. Then, the samples were analyzed using 5 % Native PAGE electrophoresis. The stability of DNA nanostructure was determined according to the formula: DNA residues (%) = (band gray value of DNA samples in RPMI-1640 medium containing 10 % FBS / band gray value of pristine DNA samples).

Fluorescence measurements

The fluorescence emission spectra were recorded by F7100 Fluorescence Spectrometer (Hitachi, Japan) with 5 nm excitation and emission slits and 700 V of PMT voltage. With the excitations of FAM at 494 nm, the fluorescence emission spectra were collected in the range of 500~620 nm for FAM-labeled DNA strands. To detect the conformation changes of DNA nanotweezer in solution, FAM and BHQ1-labeled NT-Apt (1 μM) were incubated with equal amounts of the complementary strand in 1 × Tris-HCl (pH 7.4) at 37 °C for 10 min and the fluorescence emission spectra were collected.

Flow cytometry assay

To verify the membrane anchoring capability of amphiphilic DNA nanotweezer, A549 cells and HeLa cells were plated into cell culture plates (35 mm) and cultured at 37 °C for 24 h before the experiment. Cells were washed with DPBS and counted by cell counting plate. 1×10^5 cells were resuspended in 300 μL of serum-free culture medium and incubated with FAM-labeled amphiphilic DNA nanotweezer (0.1 μM) for 30 min at room temperature away from light. To investigate the cellular uptake of amphiphilic DNA nanotweezer, cells were incubated with FAM-labeled NT-Ch1-Apt or FAM-labeled NT-Ch2-Apt (0.1 μM) for 1 h, 2 h, and 3 h separately at room temperature away from light. After that, cells were collected and used for analysis (Beckman CytoFLEX, USA). About 10000 events were counted for each sample. To confirm the tunable membrane anchoring capability of NT-Ch2-Apt, HeLa cells are resuspended in 300 μL of serum-free culture medium and incubated with FAM-labeled NT-Ch2-Apt (0.1 μM) and 0.5 μM complementary strand for 30 min at room temperature away from light. After that, cells were collected and used for flow cytometry analysis (Beckman CytoFLEX, USA). About 10000 events were counted for each sample. The normalized fluorescence intensity was determined according to the formula: Normalized fluorescence intensity = average of (experimental group fluorescence intensity/control fluorescence intensity).

Confocal microscope imaging

For the lysosomes escape experiment, after seeding in 35 mm confocal dishes and culturing overnight, A549 cells were incubated with Cy5-labeled NT-Ch1-Apt or Cy5-

labeled NT-Ch2-Apt (0.3 μM) in RPMI 1640 complete culture medium for 3 h, respectively. Then, LysoTracker green was added for another 0.5 h incubation. For the fluorescence colocalization experiment, A549 cells were incubated with Cy5-labeled NT-Ch2-Lib or Cy5-labeled NT-Ch2-Apt (0.3 μM) in complete culture medium for 30 minutes, respectively. Then, membrane dye FM4-64 was added for another 10 minutes incubation. After that, cells were washed with 1 × DPBS buffer two times for confocal imaging (Leica Stellaris 5, Germany).

Cell viability assay

To test the cytotoxicity of amphiphilic DNA nanostructures, cells were incubated with samples in 100 μL complete culture medium for 48 h. The cell viability assay was conducted by following the protocol of Cell Counting Kit-8. Briefly, 100 μL of freshly prepared CCK-8 reagent was added to each well, and the cells were incubated for another 1 h. Absorbance values at 450 nm were measured using a microplate reader (PerkinElmer EnSpire, USA). Cell viability in each group well was determined according to the formula: $(A_t / A_0) \times 100$ %, where A_t is the average absorbance value of the experimental group, and A_0 is the average absorbance value of the control group.

c-Met siRNA knockdown experiment

A549 cells (2×10^5 per well) were cultured in a 6-well plates for 12 h. Then, cells were transfected by Lipofectamine 3000 (Invitrogen, USA) loaded with c-Met siRNA (30 nM, 50 nM, and 100 nM) for 48 h before collection for western blot and flow cytometry analysis. The negative siRNA was used as a control.

Western blot analysis

A549 cells were incubated with amphiphilic DNA nanotweezer (0.5 μM) for 24 h, then washed three times with ice-cold DPBS and treated with cell lysis buffer with 1 × protease inhibitor cocktail. Protein lysates were incubated on ice for 30 min and harvested with pre-cooled scrapers. The samples were centrifuged at 14,000 rpm for 30 min. The protein concentration in the supernatants was measured by Bradford assay. Then, the protein samples were added to 5 × loading buffer and denatured at 95 °C for 10 min. Equivalent protein samples were separated using 5% concentrate gel and 8% separation gel. After separating with SDS-PAGE, proteins were transferred to polyvinylidene fluoride (PVDF) membrane with a pore size of 0.45 μm (Millipore, USA) by a wet transfer cell. The membranes were blocked with non-fat dry milk (5 % wt/vol) for 1 h at room temperature and were subsequently detected by incubating the membrane with primary rabbit monoclonal c-Raf antibody (Bethyl, USA) or primary rabbit monoclonal c-Met antibody (Cell Signaling Technology, USA) at a predetermined optimal concentration in non-fat dry milk (5 % wt/vol) overnight at 4 °C. The membranes were washed three times (10 min each) in TBST and then incubated with horseradish peroxidase (HRP)-conjugated secondary antibody (Millipore, USA) for 1 h at room temperature. Following sufficient wash with TBST, the membranes were exposed to chemiluminescent HRP substrate, protein expressions were determined using a Super Signal chemiluminescence system (ECL) and photographed by automatic chemiluminescence image processing system (Tanon, China).

Cell migration assay

A549 cells were plated into 6-well culture plates and grown to confluent monolayers for 24 h. Then the monolayer A549 cells were scratched using a sterile micropipette tip and cultured for 12 h. The culture medium was removed from the plates and washed with DPBS. The cell migration was assessed by measuring the width of the cell scratch. The healing rate was calculated as the following formula: (original scratch width - scratch width after healing) / (original scratch width) × 100 %. The images were acquired by using an inverted fluorescence microscope (Olympus IX73, Japan).

***In vivo* tumor imaging**

Female Balb/c nude mice (4 weeks) were obtained from Hunan SJA Laboratory Animal Co., Ltd. (Changsha, China). All animal experiments were approved by the Animal Care and Use Committee of Hunan University (HNU-IACUC-2023-104). To obtain a subcutaneous tumor model, 1×10^7 A549 cells in 200 μL DPBS were subcutaneously injected into the right axilla of each female mouse. Tumors were then allowed to grow over 3 weeks until the tumor grew to 200-300 mm^3 ($V = L \times W^2 \times 1/2$ (V, volume; L, length; W, width of tumor). Then, tumor-bearing BALB/c nude mice were anesthetized to be motionless with both tranquilizer and anesthetic before 200 μl 1 μM of Cy5-labeled DNA arm strand, Cy5-labeled NT-Ch2-Lib, and Cy5-labeled NT-Ch2-Apt separately was injected via the tail vein. At the preassigned time points, fluorescence images of live mice were acquired by an IVIS Lumina III system (excitation filter: 620 nm, emission filter: 670 nm) (Calip LiveScience, USA). Then,

the mice were sacrificed to collect the tumor tissues and major organs for *ex vivo* fluorescence imaging.

In vivo antitumor activity assay

4-week-old female Balb/c nude mice (Hunan SJA Laboratory Animal Co., Ltd.) received a subcutaneous injection of 1×10^7 A549 cells in 200 μL DPBS at the right axilla. Tumors were then allowed to grow for 2 weeks, and tumor size reached 50-100 mm^3. The day was set as day 0. Then, these A549 tumor-bearing mice were randomly classed into 4 groups (n = 3) and injected with 200 μL PBS, NT-Ch1-Apt, NT-Ch2-Lib, and NT-Ch2-Apt separately at tail vein every 3 days for three treatments. The dosage of each injection was 3.5 mg/kg per mice. The tumor volume and the weight of mice were recorded every other day by using a vernier caliper and electronic balance. Tumor volume was calculated as $V = L \times W^2 \times 1/2$ (V, volume; L, length; W, width of tumor). At the end of the treatment, the mice were sacrificed, and the tumors and the main organs were harvested for histological examination. The tumor growth inhibition (TGI) was calculated according to the following formula: TGI = (1 − mean tumor weight of the experimental group / mean tumor weight of the control group) × 100 %.

HE staining analysis of tissue sections

The paraffin-embedded tissue sections were immersed in xylene 2 times at 65 °C for 10 min each, rehydrated in ethanol at concentration gradient (100 %, 95 %, 85 %, 75 %) for 3 min each time, and soaked twice in ultrapure water for 3 min each time. Subsequently, the sections were stained with Hematoxylin dye for 1 min and washed

in ultrapure water to remove the floating color. Next, the slides were differentiated with differentiation solution for 30 s, and immersed 2 times in ultrapure water, for 3 min each. Then, the slides were placed in Eosin dye staining for 1 min and then immersed in ethanol solution sequentially (75 %, 85 %, 95 %, 100 %) for rapid dehydration. After that, the slides were washed with 100 % ethanol for 1 min, then soaked in xylene 2 times, for 1 min each, and finally sealed with neutral gum. Slides were analyzed using a digital section scanning system (Pannoramic MIDI, 3DHISTECH Ltd., Hungary).

Statistical analysis

Bar and line plots are represented as mean ± s.d. Paired Student's t-test was used to calculate statistical significance. Statistical analysis was conducted using Prism 8.0 (GraphPad, San Diego, CA, USA). A value of $P \leqslant 0.05$ was considered statistically significant.

Safety

No unexpected or unusually high safety hazards were encountered in this work.

3. Supplementary Tables

Table S1. DNA sequences used in this study.

Name	Sequence information (5'→ 3')
c-Met Apt	TGG ATG GTA GCT CGG TCG GGG TGG GTG GGT TGG CAA GTC T
FAM-c-Met	FAM-TGG ATG GTA GCT CGG TCG GGG TGG GTG GGT TGG CAA GTC T
Library strand	ACC TGC CAT GCT CGG TCG GGG TGG GTG GGT TGC GTT CAG A
DNA arm strand 1	GGT GTC ACA GGC GGG ACC CCC GAC CGA
FAM-DNA arm strand 1	FAM-GGT GTC ACA GGC GGG ACC CCC GAC CGA
Cy5-DNA arm strand 1	Cy5-GGT GTC ACA GGC GGG ACC CCC GAC CGA
ASO	GTC CCG CCT GTG ACA CCT CCC GCC TGT GAC ATG CAT TTT TTT TCC CGC CTG TGA CAT GCA TTG TCC CGC CTG TGA CAC C
FAM-ASO-BHQ1	FAM-GTC CCG CCT GTG ACA CCT CCC GCC TGT GAC ATG CAT TTT TTT TCC CGC CTG TGA CAT GCA TTG TCC CGC CTG TGA CAC C-BHQ-1
ASO-Ch1	Chol-GTC CCG CCT GTG ACA CCT CCC GCC TGT GAC ATG CAT TTT TTT TCC CGC CTG TGA CAT GCA TTG TCC CGC CTG TGA CAC C
ASO-Ch2	Chol-GTC CCG CCT GTG ACA CCT CCC GCC TGT GAC ATG CAT TTT TTT TCC CGC CTG TGA CAT GCA TTG TCC CGC CTG TGA CAC C-Chol
DNA arm strand 2	CAA CCC ACC CGG TGT CAC AGG CGG GAC
Complementary strand	AGA CTT GCC AAC CCA CCC ACC CCG ACC GAG CTA CCA TCC A
Noncomplementary strand	ACA ACA GCC TCA AGA TCA TC
c-Met siRNA sense	GUG CAG UAU CCU CUG ACA GUU
c-Met siRNA antisense	CUG UCA GAG GAU ACU GCA CUU

4. Supplementary Figures

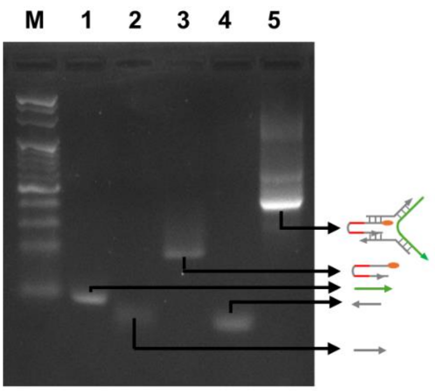

Figure S1. Characterization of NT-Ch1-Apt by 3 % agarose gel electrophoresis. From lanes 1 to 5: c-Met aptamer, DNA arm strand 1, ASO-Ch1, DNA arm strand 2, NT-Ch1-Apt. M: Marker.

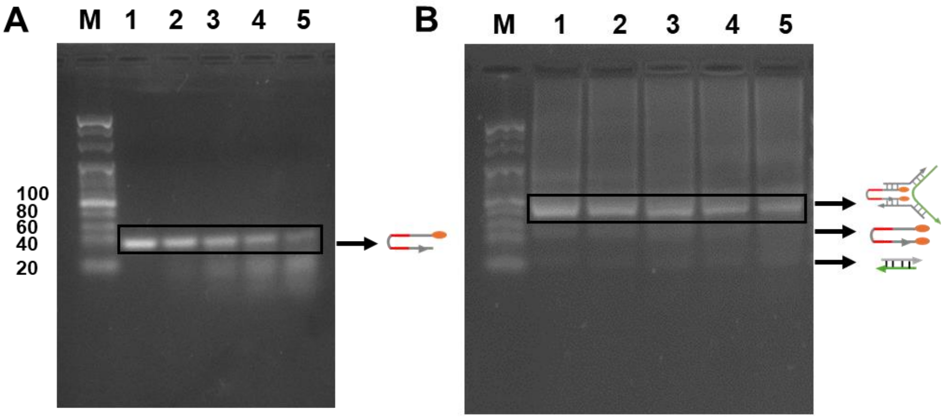

Figure S2. Stability analysis of ASO-Ch1 (A) and NT-Ch2-Apt (B) in RPMI1640 medium supplemented with 10 % FBS by agarose gel electrophoresis (3 %). From lanes 1 to 5: 0 h, 1 h, 3 h, 6 h, 12 h. M: Marker.

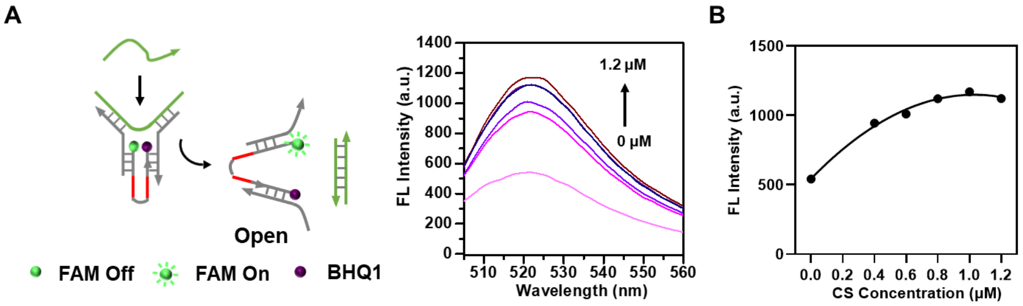

Figure S3. (A) The fluorescence spectra of FAM and BHQ1-labeled NT-Apt incubated with complementary strand with various concentrations (from 0-1.2 μM). (B) The resultant linear relationship plotted by the fluorescence intensity at 521 nm vs the various concentration of complementary strand.

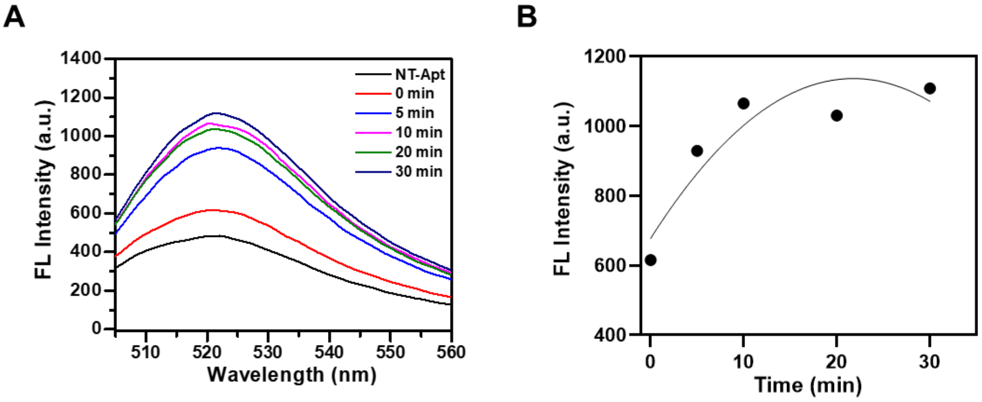

Figure S4. (A) The fluorescence spectra of FAM and BHQ1-labeled NT-Apt incubated with complementary strand (1 μM) were tracked over time. (B) The resultant linear relationship plotted by the fluorescence intensity at 521 nm vs the various incubation time.

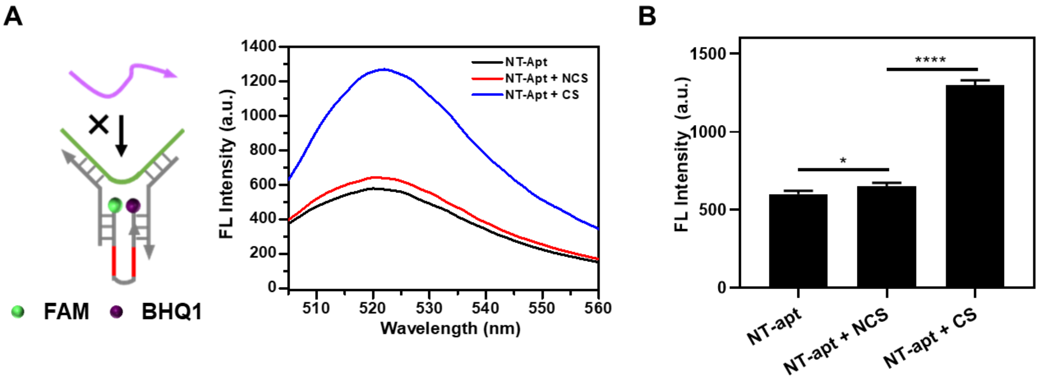

Figure S5. (A) The fluorescence spectra of FAM and BHQ1-labeled NT-Apt incubated with complementary strand (CS) and noncomplementary strand (NCS) separately. (B) Quantitative results of the averaged fluorescence intensity of different treatment groups (*$P \leqslant 0.05$, ****$P \leqslant 0.0001$).

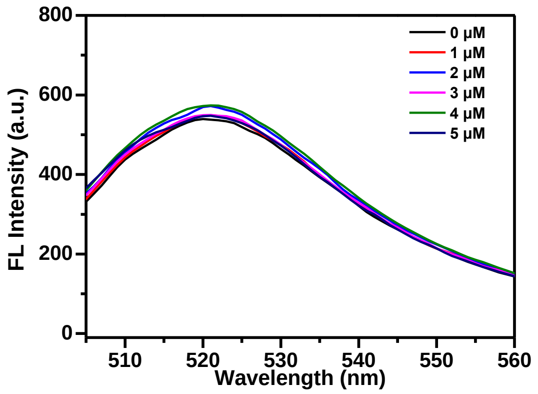

Figure S6. The fluorescence spectra of FAM and BHQ1-labeled NT-Apt incubated with noncomplementary strand with various concentrations (from 0-5 μM).

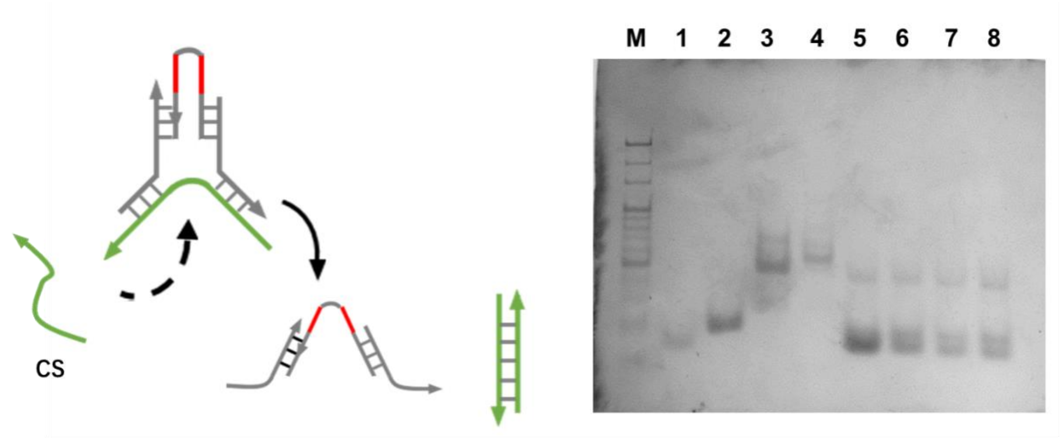

Figure S7. PAGE (5 %) analysis of strand displacement reaction of NT-Apt with complementary strand (CS). From Lanes 1 to 4: complementary strand, the DNA duplex of complementary strand and c-Met aptamer, NT, NT-Apt; from Lanes 5 to 8: strand displacement reaction of NT-Apt with complementary strand for 15 min,30 min, 45 min, and 60 min, separately. M: Marker.

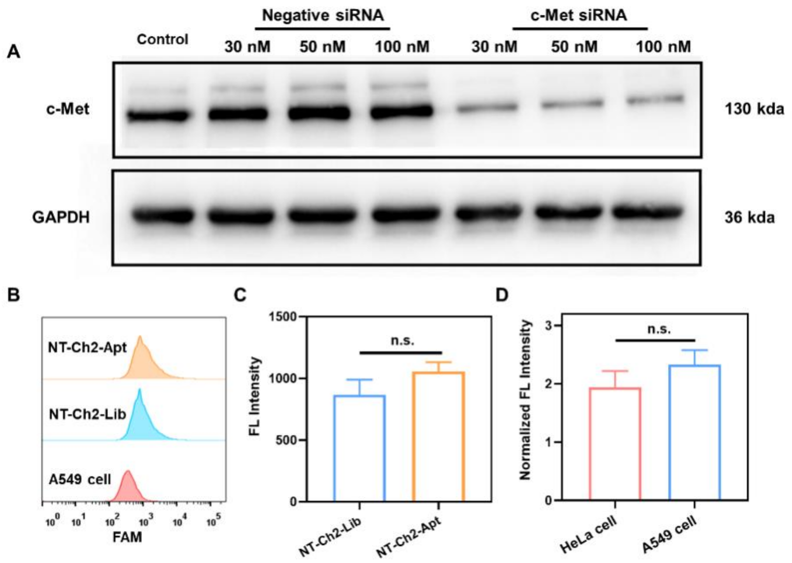

Figure S8. (A) Western blot analysis of c-Met expression in A549 cells treated with c-Met specific siRNA at indicated concentrations (30 nM, 50 nM, and 100 nM) for 48 h. (B) Flow cytometry analysis of c-Met knockdown A549 cells incubated with FAM-labeled NT-Ch2-Lib or FAM-labeled NT-Ch2-Apt for 30 minutes separately. (C) Quantitative results of the averaged fluorescence intensity of c-Met knockdown A549 cells in different treatment groups in (B). (D) Normalized fluorescence intensity of nontarget HeLa cells and c-Met knockdown A549 cells incubated with FAM-labeled NT-Ch2-Apt for 30 minutes separately.

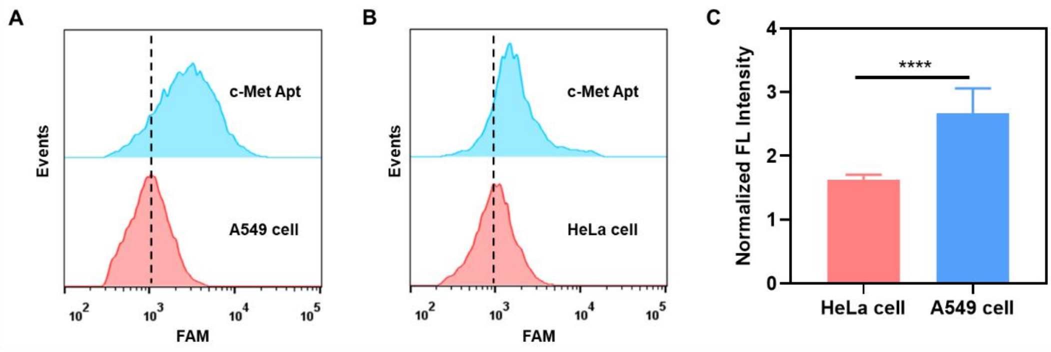

Figure S9. Flow cytometry analysis of A549 cells (A) or HeLa cells (B) incubated with FAM-labeled c-Met aptamer separately. (C) Normalized the averaged fluorescence intensity of cells incubated with FAM-labeled c-Met aptamer in (A, B) (****$P \leqslant 0.0001$).

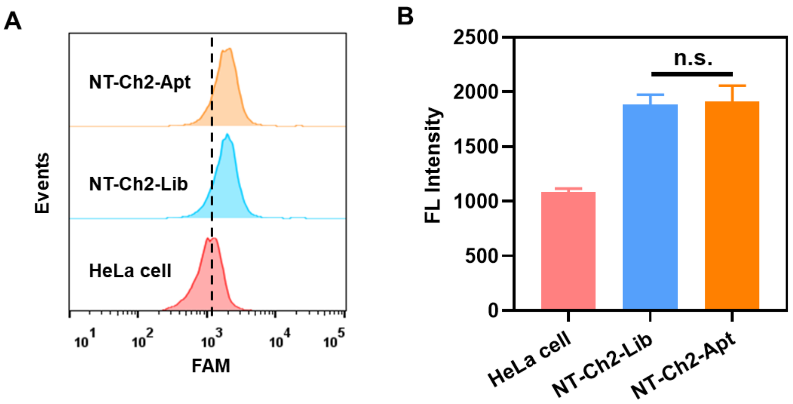

Figure S10. (A) Flow cytometry analysis of HeLa cells incubated with FAM-labeled NT-Ch2-Lib or FAM-labeled NT-Ch2-Apt separately. (B) Quantification of the averaged fluorescence intensity of different treatment groups in (A).

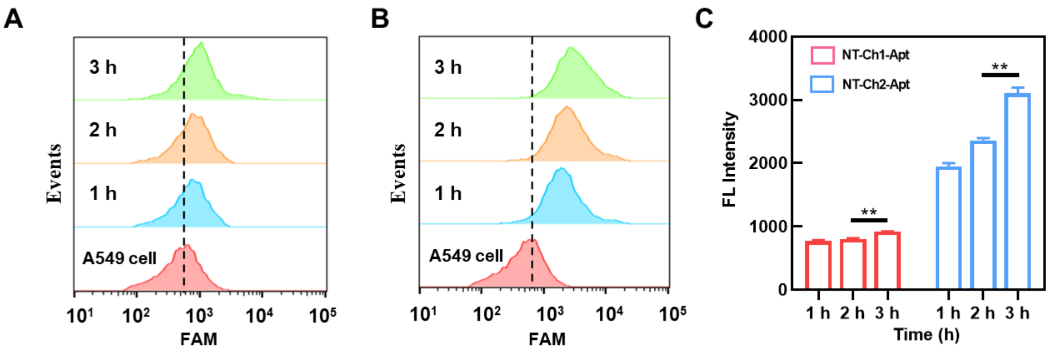

Figure S11. Flow cytometry analysis of A549 cells incubated with FAM-labeled NT-Ch1-Apt (A) or FAM-labeled NT-Ch2-Apt (B) separately at different times. (C) Quantification of the averaged fluorescence intensity of cells with different treatments in (A, B) (**$P \leqslant 0.01$).

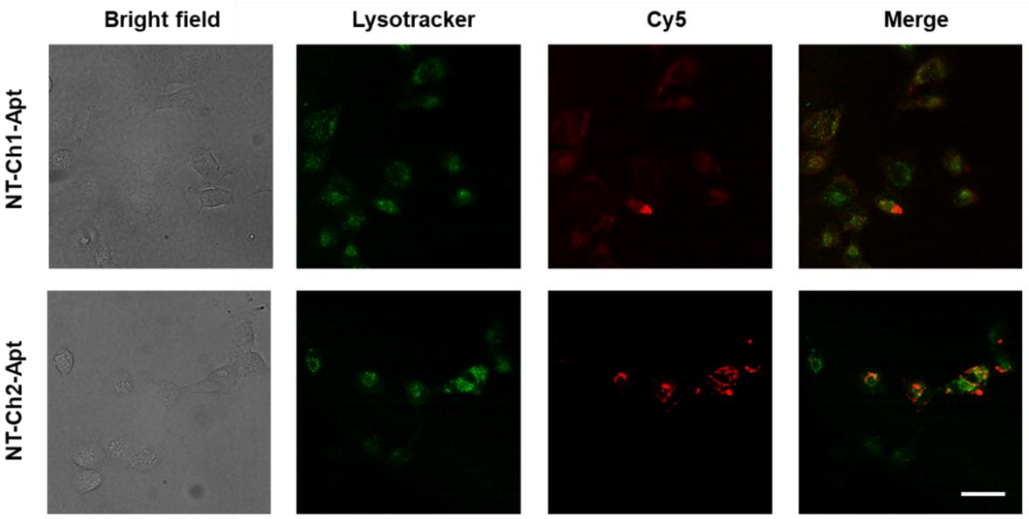

Figure S12. Representative confocal images of A549 cells incubated with Cy5-labeled NT-Ch1-Apt or Cy5-labeled NT-Ch2-Apt separately. Lysosome was labeled with Lysotracker Green. Scale bar, 50 μm.

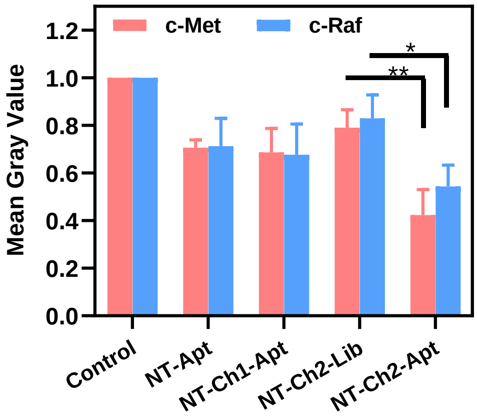

Figure S13. Western blot analysis of c-Met and c-raf expression in A549 cells incubated with indicated samples for 24 h and untreated cells are used as control (*$P \leqslant 0.05$, **$P \leqslant 0.01$).

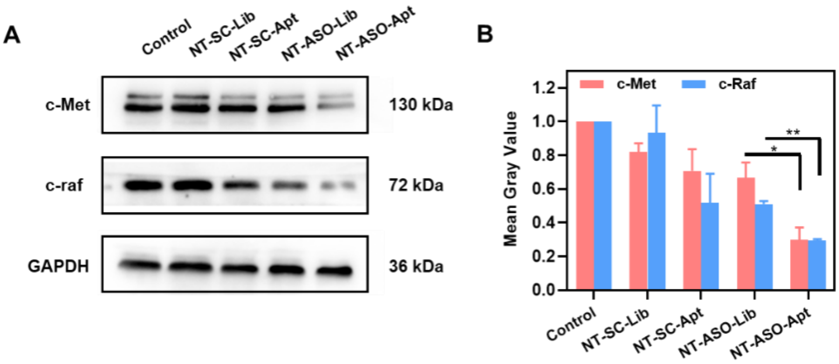

Figure S14. (A) Western blot analysis of c-Met and c-raf protein in A549 cells treated with NT-SC-Lib, NT-SC-Apt, NT-ASO-Lib, and NT-ASO-Apt separately for 24 h and untreated cells are used as control. (B) Quantification of the grayscale value of (A). (*$P \leqslant 0.05$, **$P \leqslant 0.01$).

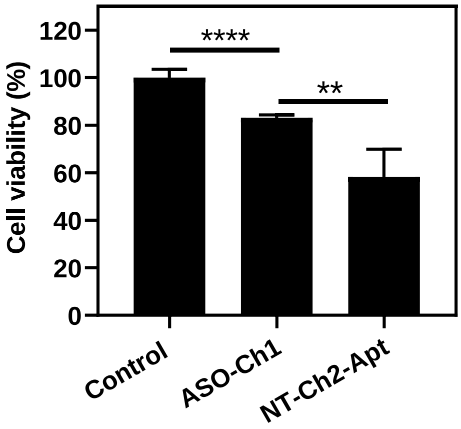

Figure S15. Cell viability assay of A549 cells incubated with amphiphilic DNA nanostructures (500 nM) for 48 h and untreated cells are used as control (** $p \leqslant 0.01$, **** $p \leqslant 0.0001$).

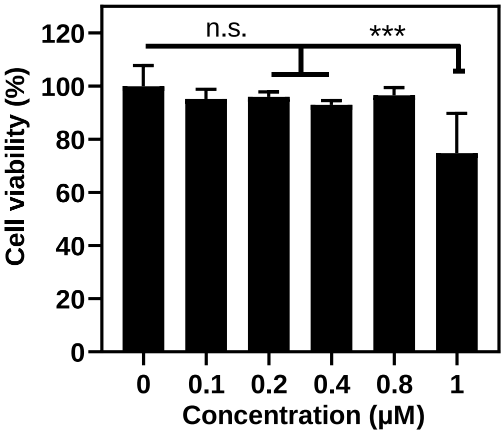

Figure S16. Cell viability assay of HeLa cells incubated with NT-Ch2-Apt (0-1 μM) for 48 h and untreated cells are used as control. (*** $P \leqslant 0.001$).

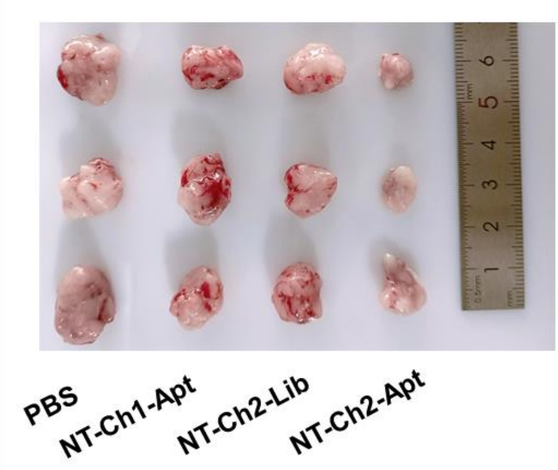

Figure S17. Photographs of tumors dissected from A549 tumor-bearing mice after indicated treatments.

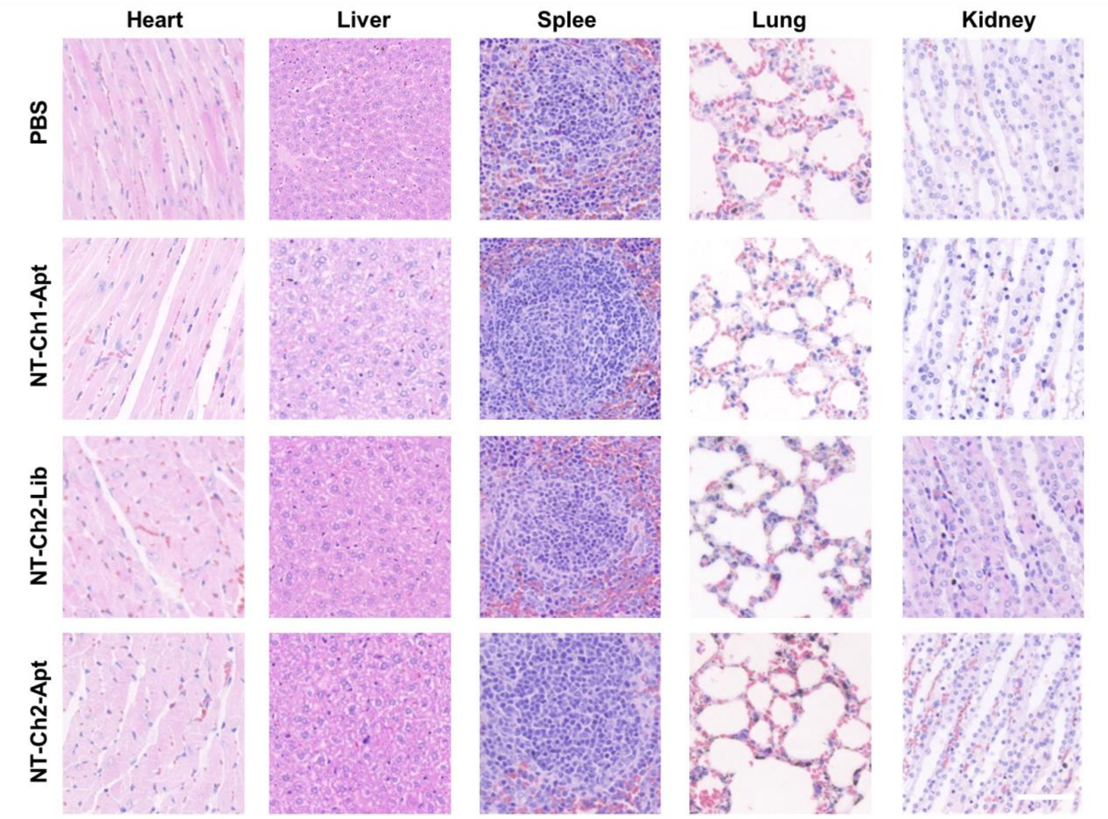

Figure S18. Hematoxylin-eosin (H&E) staining of the major organs derived from the A549 tumor-bearing mice after indicated treatments. Scale bar, 50 μm.
